# Supplementary material for: Complexome profiling of the Chlamydomonas psb28 mutant reveals TEF5 as an early PSII assembly factor
Source: Plant Cell. 2025 Mar 17;37(6):koaf055. doi: 10.1093/plcell/koaf055 (PMC12164590; doi:10.1093/plcell/koaf055)
Supplement: koaf055_Supplementary_Data [file koaf055_supplementary_data.zip › psb28_interactive.zip!/plots/intensity/qualityPlots/peptides/ATPVD2_Cre09.g405050.t1.2.html]

### Cre09.g405050.t1.2

Trivial name: ATPVD2  
SeqCoverage [median]: 25.30  
Mass: 29.67 kDa  
  
 

| Sequence | MissedCleavage | Mass [Da] | wtIntensity | muIntensity | Score |
| --- | --- | --- | --- | --- | --- |
| AELDELEREEFFR | 1 | 1681.79 | 2285.86 | 3628.36 | 47.29 |
| FESFVLPGETK | 0 | 1252.63 | 0.00 | 5923.76 | 122.26 |
| GDLDNVAGVK | 0 | 986.50 | 0.00 | 562.36 | 60.59 |
| GDLDNVAGVKIPR | 1 | 1352.74 | 0.00 | 1348.89 | 51.01 |
| LENTISYIK | 0 | 1079.59 | 1856.58 | 7684.37 | 128.36 |
| RVNALENVVKPK | 2 | 1365.81 | 0.00 | 855.20 | 82.17 |
| VRGDLDNVAGVK | 1 | 1241.67 | 1876.97 | 994.90 | 85.85 |
|  |  |  | 6019.4 | 20997.8 |  |
